# Solid-Contact Potentiometric Sensors Based on Main-Tailored Bio-Mimics for Trace Detection of Harmine Hallucinogen in Urine Specimens

**DOI:** 10.3390/molecules26020324

**Published:** 2021-01-10

**Authors:** Abde El-Galil E. Amr, Ayman H. Kamel, Abdulrahman A. Almehizia, Ahmed Y. A. Sayed, Hisham S. M. Abd-Rabboh

**Affiliations:** 1 Pharmaceutical Chemistry Department, College of Pharmacy, King Saud University, Riyadh 11451, Saudi Arabia; aamr@ksu.edu.sa; 2 Applied Organic Chemistry Department, National Research Center, Dokki, Giza 12622, Egypt; mehizia@ksu.edu.sa (A.A.A.); ahmedyahia009@gmail.com (A.Y.A.S.); 3 Department of Chemistry, Faculty of Science, Ain Shams University, Cairo 11566, Egypt; 4 Chemistry Department, Faculty of Science, King Khalid University, P.O. Box 9004, Abha 61413, Saudi Arabia

**Keywords:** solid-contact, poly(3,4-ethylenedioxythiophene) (PEDOT), harmine, hallucinogen, ion-selective membrane sensors, imprinted polymers

## Abstract

All-solid-state potentiometric sensors have attracted great attention over other types of potentiometric sensors due to their outstanding properties such as enhanced portability, simplicity of handling, affordability and flexibility. Herein, a novel solid-contact ion-selective electrode (SC-ISE) based on poly(3,4-ethylenedioxythiophene) (PEDOT) as the ion-to-electron transducer was designed and characterized for rapid detection of harmine. The harmine-sensing membrane was based on the use of synthesized imprinted bio-mimics as a selective material for this recognition. The imprinted receptors were synthesized using acrylamide (AA) and ethylene glycol dimethacrylate (EGDMA) as functional monomer and cross-linker, respectively. The polymerization process was carried out at 70 °C in the presence of dibenzoyl peroxide (DBO) as an initiator. The sensing membrane in addition to the solid-contact layer was applied to a glassy-carbon disc as an electronic conductor. All performance characteristics of the presented electrode in terms of linearity, detection limit, pH range, response time and selectivity were evaluated. The sensor revealed a wide linearity over the range 2.0 × 10^−7^–1.0 × 10^−2^ M, with a detection limit of 0.02 µg/mL and a sensitivity slope of 59.2 ± 0.8 mV/hamine concentration decade. A 40 mM Britton–Robinson (BR) buffer solution at pH of 6 was used for all harmine measurements. The electrode showed good selectivity towards harmine over other common interfering ions, and maintained a stable electrochemical response over two weeks. After applying the validation requirements, the proposed method revealed good performance characteristics. Method precision, accuracy, bias, trueness, repeatability, reproducibility, and uncertainty were also evaluated. These analytical capabilities support the fast and direct assessment of harmine in different urine specimens. The analytical results were compared with the standard liquid chromatographic method. The results obtained demonstrated that PEDOT/PSS was a promising solid-contact ion-to-electron transducer material in the development of harmine-ISE. The electrodes manifested enhanced stability and low cost, which provides a wide number of potential applications for pharmaceutical and forensic analysis.

## 1. Introduction

Harmine (7-methoxy-1-methyl-9H-pyrido [3,4,6]indole), known as banisterine, is a fluorescent harmala alkaloid. It is a member of the beta-carboline family. It can be found in many organisms, including plants and butterflies. Tobacco, *Peganum harmala* (widely in Middle Eastern) and lemon balm are listed as natural sources of harmine [1,2,3]. Harmine is an inhibitor of monoamine oxidase (MAOIs) and antagonist of 5-hydroxytryptamine (5-HT) [4,5,6]. It reversibly binds to MAO-A and inhibits the activity of monoamine oxidase enzymes, increasing the concentration of neurotransmitters norepinephrine, serotonin and dopamine from the brain. Therefore, harmine was used as an antidepressant [4,5,6]. Other studies revealed that harmine has many other functions including anti-cancer effects [7], inducing pancreatic islet cell proliferation [8]. It can be added to different hallucinogenic drinks in different countries such as yage in Columbia, ayahuasca in Ecuador and Bolivia, and caapi in Brazil [9]. Injecting harmine into a vein can cause hallucinations [10]. In addition, it has been found that harmine has anti-worm activity by paralyzing the tension of the muscles of the parasites. So, it is commonly used internally as a treatment for trap worms [11].

Different techniques were reported in the literature for carboline alkaloids analysis in biological samples. Some of these techniques are high performance liquid chromatography (HPLC) coupled with electrochemical [12], photodiode array (DAD) [13], fluorescence [14,15] or mass spectrometry (MS) detectors [16], as well as gas chromatography (GC–MS) [17]. Most of these methods lack simplicity in use, require very sophisticated instruments, need well trained personnel, and require several time-consuming manipulation steps. Electrochemical techniques offer fast analysis, low-cost instrumentation, capability for simultaneous determination, high sensitivity and a remarkably low detection limit. Recently, potentiometric sensors have attracted great attention as important and reliable devices for the analysis in different fields such as chemical, pharmaceutical, forensic and biomedical analyses [18,19,20,21,22,23,24,25,26,27,28,29]. One potentiometric poly(viny1 chloride) matrix membrane sensor was reported in the literature for harmine assessment [30]. The method was not applicable in the determination of harmine in biological fluids and presented bad selectivity over some other alkaloids such as Cinchonine, Quinine and Brucine.

All-solid-state potentiometric sensors have attracted great attention over other types of potentiometric sensors due to their outstanding properties such as enhanced portability, simplicity of handling, affordability and flexibility [20,21,22]. They can be considered now as the next generation for electrochemical sensors based on potentiometric transduction. A lipophilic solid-contact material is placed at the interface between the sensing-membrane and the electronic conductor (EC) substrate to eliminate the unstable boundary potential response, which is produced because of the unfavorable blocked interface between the ion-sensing membrane (ISM) and EC. This can be assigned as the essential part for designing robust and reliable solid-state ion-selective electrodes (ISEs). In addition, this enhances the long-term potential stability and the reproducibility of these types of sensors.

Man-tailored bio-mimics “molecularly-imprinted polymers (MIPs)” are well known as good receptors for different templated targets. Recently, MIPs were successfully integrated in the design of all types of potentiometric sensors [31,32,33,34]. They successfully shifted the view of using non-affordable ionophores. These ionophores were limited by their high-cost or using ion- exchangers, which revealed poor selectivity behavior. These man-made receptors are featured by their enhanced thermal stability, ease of preparation and their low-cost [35]. Recently, potentiometric ISEs based on MIPs were integrated in designing different ISEs that were used for the quantification of different templated organic molecules [36,37,38].

Herein, we present a novel system with PEDOT/PSS-doped used as an ion-to-electron transducer in ion-selective electrodes (ISEs) for monitoring harmine in urine specimens. The sensing membrane is based on the use of the ion-association complexes of the harminium cation with 5-nitrobarbaturate as a recognition material for the recognition. Reproducibility, conditioning, and potential-stability were investigated. The performance characteristics of the presented sensors such as linearity response, selectivity, detection limit and selectivity were also evaluated. The sensors were applied for monitoring harmine hallucinogen in urine specimens, and the results were compared with the standard gas-liquid chromatography method [39].

## 2. Results and Discussions

### 2.1. Polymers’ Characterizations

**Figure 1:**
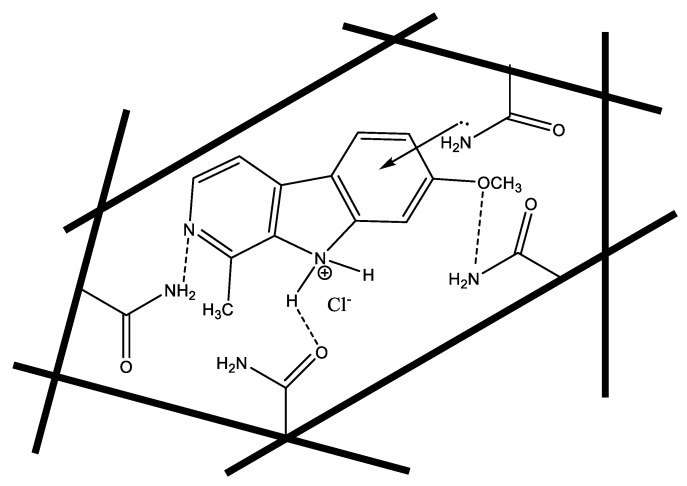
Non-covalent harmine imprinting.

The imprinting approach that was used for MIPs preparation of harmine was the non-covalent molecular imprinting method (Figure 1). The amino, methoxy and N-pyridine groups of the template harmine can form strong hydrogen bonding with the functional monomer acrylamide (AA). In addition, there is a charge-transfer complex interaction that can take place between the electron-deficient aromatic ring in harmine and the electron-rich amino group of the AA monomer [40,41].

**Figure 2:**
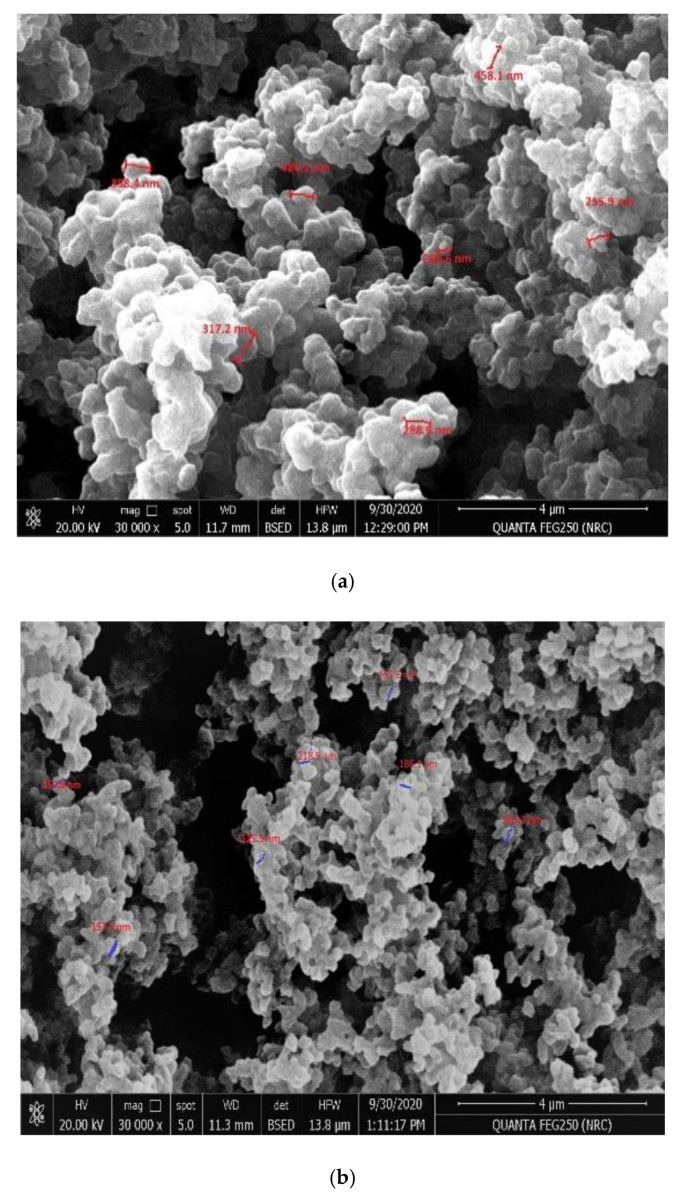
SEM images of (**a**) MIPs and (**b**) NIPs; [4 µm; 30,000×; 20.0 kV].

As shown in Figure 2, the scanning electron microscope of either MIPs or non-imprinted polymers (NIPs) nano-beads can present a picture for the surface morphologies of the obtained polymers. The MIPs beads showed a semi-uniformed spherical shape with a size distribution of 255.8–486.2 nm, whereas the NIPs particles were of irregular and much smaller (a size distribution of 125.5–218.9 nm). This can indicate that the presence of harmine as a templated molecule has a great influence on the shape of the polymer formed, in which the template-monomer complex can change the solubility of the growing polymer. This has a great influence on altering the polymer morphology [42,43].

### 2.2. Membrane Optimization

These artificial receptors when dispersed in plasticized polyvinyl chloride (PVC)-membranes provided highly sensitive and selective sensors for harmine monitoring. Solid-contact sensors (i.e., without internal filling solution), made of glassy carbon (GC)-disks coated with the membrane cocktail were prepared, characterized and examined for determining harmine. Four membrane sensors for sensor type were designed and characterized during 8 weeks according to IUPAC recommendations [44].

**Figure 3:**
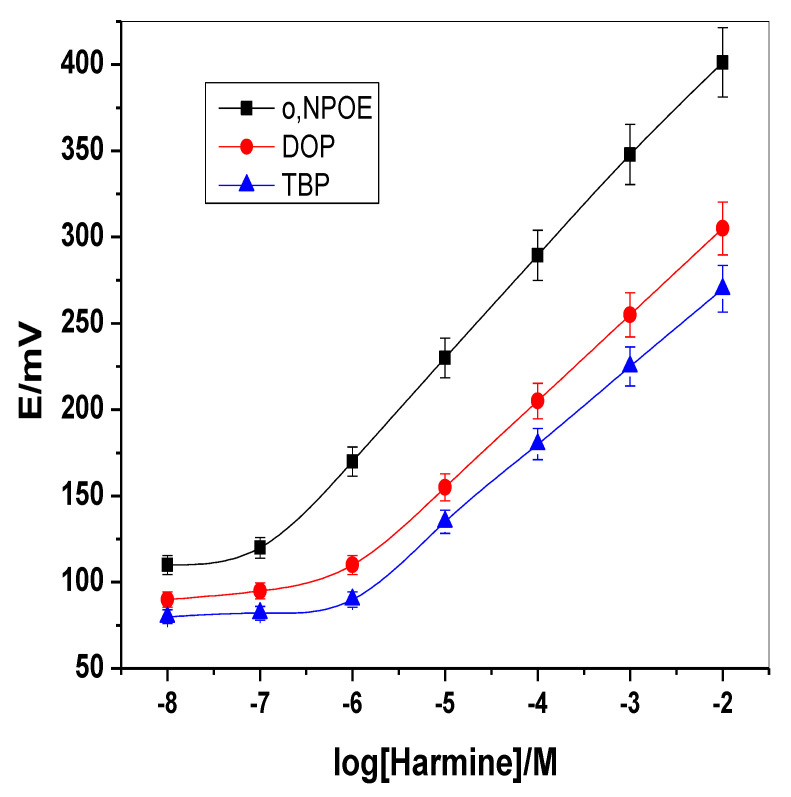
Effect of solvent mediator polarity on the potentiometric response of harmine membrane-based sensor.

For membrane optimization, the potentiometric response of the sensor is greatly influenced by the polarity of the membrane medium. Harmine-based sensor incorporating MIP nano-beads with tetrabutyl phosphate (TBP), dioctyl phosphate (DOP) and o-nitrophenyloctyl ether (o-NPOE) plasticizers were designed and checked. The calibration slope and detection limit were found to be 58.0 ± 0.7, 50.1 ± 0.3 and 45.2 ± 0.6 mV/decade and 0.03, 0.23 and 0.68 µg/mL upon using o-NPOE (*ε* = 24) instead of DOP (*ε* = 7) and TBP (*ε* = 4), respectively. As shown in Figure 3, the membranes incorporating o-NPOE revealed a more favorable calibration slope and lower limit of detection than those containing either DOP or TBP plasticizer. As a control, harmine-ISEs based on NIP nano-beads were also characterized. The electrodes based on NIP revealed a sub-Nernstian slope of 19.6 ± 0.9 mV/decade (*R^2^* = 0.998) with a linearity range 5.0 × 10^−5^–1.0 × 10^−2^ M of harmine solution and a detection limit of 8.0 × 10^−5^ M. All potentiometric features for the presented sensors were shown in Table 1.

The selectivity coefficients of membrane sensors with different plasticizers were shown in Table 2. Selectivity for harmine in presence of other common organic cations such as harmaline, strychnine, cinchonine, quinine, brucine, adrenaline and caffeine were enhanced with o-NPOE membrane-based sensors. All subsequent measurements were carried out using membranes-based o-NPOE plasticizer.

In the presence of PEDOT/PSS as an ion to electron transducer and o-NPOE plasticizer, the sensor revealed a Nernstian slope of 59.2 ± 0.8 mV/decade (*n* = 5, *r*^2^ = 0.9996) and detection limit of 0.02 µg/mL. This confirmed that PEDOT/PSS layer has no influence on the potentiometric response of the sensor.

### 2.3. Time Response and pH Effect

**Figure 4:**
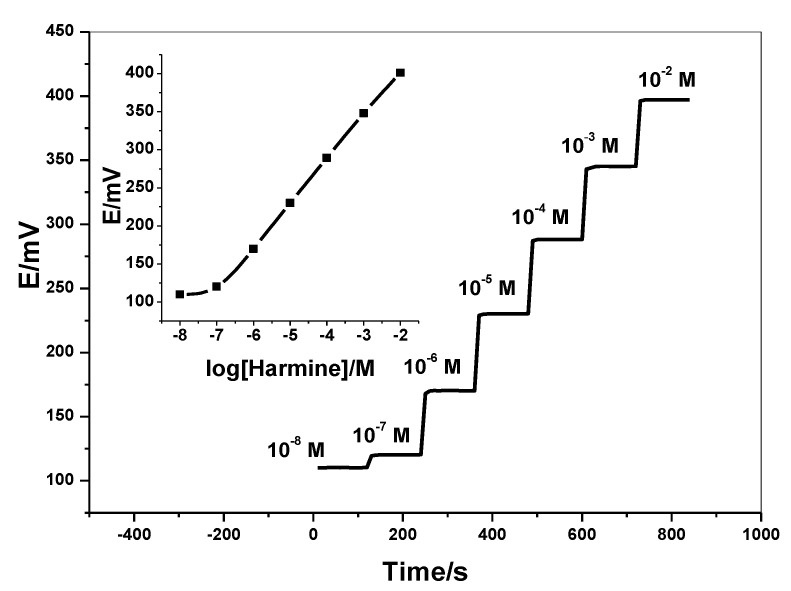
Time response of C/PEDOT:PSS/harmine-ISE (inset: the calibration plot of the sensor).

The time response of C/PEDOT:PSS/harmine-ISE was shown in Figure 4. The steady time attaining equilibrium is recorded in 1.0 × 10^−8^–1.0 × 10^−2^ M harmine solutions. The time was found to be <10 s after a 10-fold rapid increase in concentration. The results confirmed that the presented electrode has high stability and it can be used as a rapid and automated analytical tool. Repeatability was evaluated by using 40 mM Britton–Robinson (BP) buffer, pH 6 over 2.5 h. No remarkable change in the response was observed during this interval. After re-calibration for 10 times/day, it was noticed that no significant change in the recorded slope 59.2 ± 0.8 mV/decade (*n* = 6) and limit of detection 0.02 µg/mL (*n* = 10) which reflects the high reproducibility of the presented sensor.

The effect of pH on the potentiometric response of C/PEDOT:PSS/harmine-ISE was checked over the pH range 2–10. The sensor revealed a constant potential response over the pH range 3–7.5, which can be taken as the working pH range for harmine assessment. At pH values > 8, the potential response of the sensor declined due to the formation of the un-protonated harmine base, which cannot be detected by the sensor. An increase in the potential response at pH values < 3 due to positive interference by H^+^ ions. Therefore, 40 mM Britton-Robinson (BR) buffer solution, pH 6 was chosen for all subsequent measurements. 

### 2.4. Selectivity Behavior

The selectivity behavior of harmine sensors was tested towards different alkaloids, amines and inorganic cations. The selectivity coefficient values expressed as (Log *K^pot^_Harmine, J_*) were calculated and are presented in Table 3. The results revealed that harmine sensors exhibited a good selectivity in the presence of many basic organic compounds. Addition of PEDOT/PSS layer has no effect on the potentiometric selectivity pattern. This is clear from the selectivity coefficient values obtained by C/PEDOT:PSS/harmine-ISE and C/harmine-ISE. The presented electrodes showed significant enhanced selectivity other than the selectivity presented by Hassan et al. [30]. This reflects the successful use of MIPs as ionophores for harmine.

### 2.5. Impedance Spectroscopy and Chronpotentiometry Measurements

**Figure 5:**
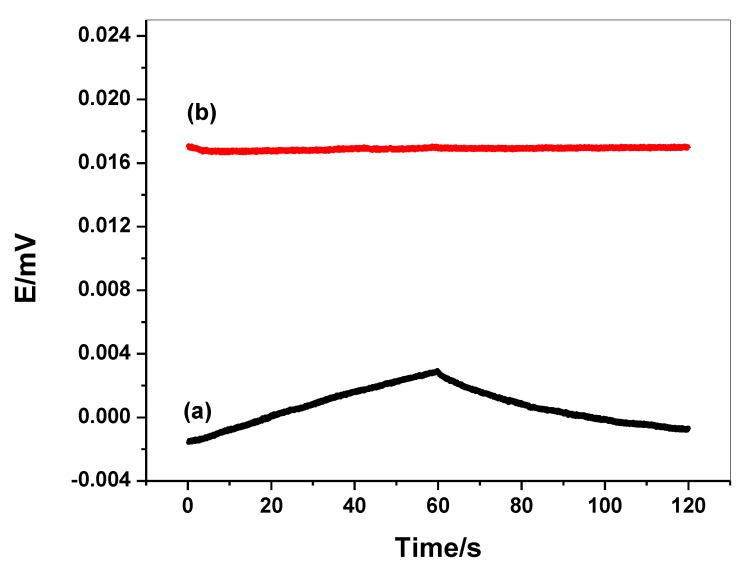
Chronopotentiogram for (**a**) C/harmine-ISE; and (**b**) C/PEDOT:PSS/harmine-ISE.

The chronopotentiograms (*E* versus *t* plots) were shown in Figure 5. The chronopotentiograms showed the potential-jump (Δ*E*) after changing the current-direction and a potential drift (Δ*E*/Δ*t*) at longer times. In addition, this potential jump was used to calculate the total resistance (*R_b_*) of the presented sensor (Δ*E* = *I* · *R_b_*), which is controlled by the bulk resistance of the ISM. The results of *R_b_* were found to be (0.23 ± 0.02 MΩ) and (0.21 ± 0.05 MΩ) for both C/PEDOT:PSS/harmine-ISE and C/harmine-ISE, respectively. The potential drift (Δ*E*/Δ*t*) of C/PEDOT:PSS/harmine-ISE (1.37 ± 0.5 µV/s) was found to be lower than C/harmine-ISE (63.3 ± 1.3 µV/s). The redox capacitance (*C* = *I*/(Δ*E*/Δ*t*)) was calculated and found to be 729.9 ± 4.5 and 15.8 ± 1.3 µF for C/PEDOT:PSS/harmine-ISE and C/harmine-ISE, respectively. The results indicated an enhanced potential stability and high capacitance for the presented sensor upon the addition of PEDOT/PSS as a solid contact material.

**Figure 6:**
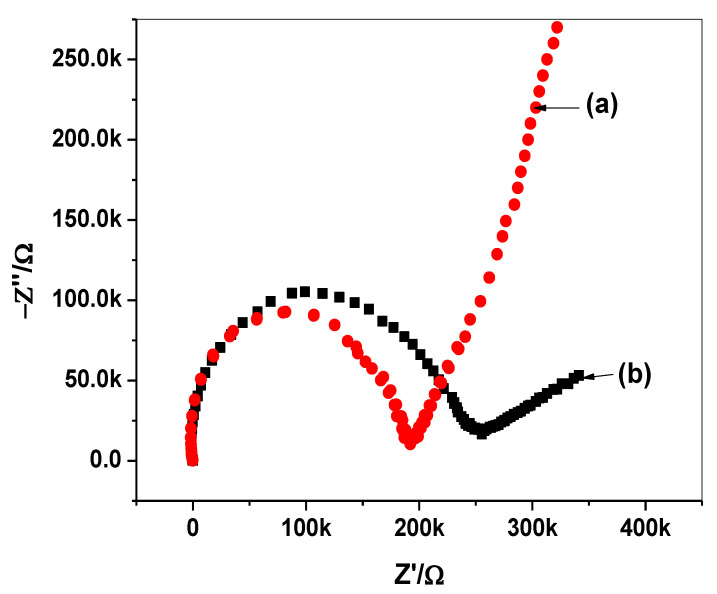
Impedance spectra for (**a**) C/harmine-ISE and (**b**) C/PEDOT:PSS/harmine-ISE.

Impedance measurements were carried out for both C/PEDOT:PSS/harmine-ISE and C/harmine-ISE. The impedance spectra were recorded at the open-circuit potential in 10 mM HMR solution and are shown in Figure 6. From the high-frequency semicircle part, the total resistance (R) was found to be 0.25 ± 0.03 and 0.18 ± 0.04 MΩ for both C/PEDOT:PSS/harmine-ISE and C/harmine-ISE, respectively. The capacitance (*C*) for either C/PEDOT:PSS/harmine-ISE or C/harmine-ISE was estimated from the low-frequency semicircle part. The capacitance (*C*) was found to be 722.6 ± 3.5 and 13.8 ± 0.7 µF for either C/PEDOT:PSS/harmine-ISE or C/harmine-ISE, respectively. The obtained results confirmed that the lipophilic character of PEDOT/PSS layer can generate a large redox capacitance, which is responsible for the enhanced potential stability of the presented ISEs.

### 2.6. Water-Layer Test

**Figure 7:**
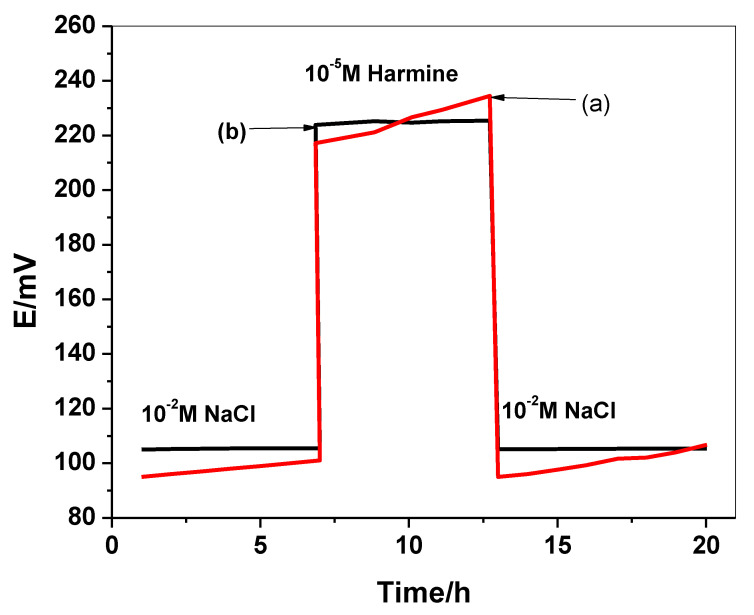
Water-layer test for (**a**) C/harmine-ISE and (**b**) C/PEDOT:PSS/harmine-ISE.

A severe potential drift can arise in the ISE because of the existence of the water-layer between the ISM and the electronic substrate. This potential drift can be eliminated after the insertion of a hydrophobic solid-contact layer at the interface between the ISM and the electronic conductor. The water-layer test is performed to distinguish the existence of this layer. The electrodes were inserted at first in 10^−2^ M NaCl for 7 h and then inserted in 10^−5^ M HMR solution for another 6 h, then changed back to 10^−2^ M NaCl solution. The potential was recorded over all these intervals. As shown in Figure 7, C/PEDOT:PSS/harmine-ISE revealed higher potential-stability than C/harmine-ISE, especially when going back to the harmine solution. This confirms the non-existence of the water-layer in the C/PEDOT:PSS/harmine-ISE after the insertion of the lipophilic PEDOT:PSS layer. The long-term stability of both C/PEDOT:PSS/harmine-ISE and C/harmine-ISE was calculated from the potential-response at the final part of the water-layer test (Figure 7). The potential drift obtained was calculated and found to be 0.1 and 4.2 mV/h for both C/PEDOT:PSS/harmine-ISE and C/harmine-ISE, respectively. This confirmed that the insertion of the PEDOT:PSS layer enhanced the potential-stability and reflected the high lipophilicity of this solid-contact material.

### 2.7. Analytical Assessment of Harmine

The presented C/PEDOT:PSS/harmine-ISE was introduced to determine harmine in urine specimens spiked with different amounts of harmine. Each sample was analyzed in triplicates and the results were in comparison with the standard liquid chromatographic method (HPLC) as a comparative technique. All results were shown in Table 4. The average recoveries varied between 94–102% and 98–105% for the presented potentiometric method and HPLC method, respectively. The *t*-student and *F*-tests data emphasized that there is no observed difference between the set of results obtained by the presented potentiometric method and the standard HPLC method. The presented potentiometric method revealed an enhanced applicability as a new methodology for harmine assessment.

## 3. Materials and Methods

### 3.1. Apparatus

“All potential values were measured by a PXSJ-216 pH/mV meter (INESA Scientific Instrument Co., Ltd., Shanghai, China). The designed electrodes were screen-printed electrodes (SPEs) purchased from DropSens Metrohm (Oviedo (Asturias, Spain) (Ceramic substrate: L33 × W10 × H0.5 mm). The working electrodes were made from carbon with 4 mm circular area. Metrohm potentiostat/galvanostat (Autolab, model 204, Herisau, Switzerland) was used for impedance and chronopotentiometric measurements. For chromatographic determination of harmine, HPLC as a reference method was carried out using HPLC (Agilent 1200, Agilent, MA, USA) coupled with a photodiode array detector. The separation was carried out on a chromatographic column (GraceSmart RP-18 (6.0 mm × 150 mm × 5 µm), MZ-Analsentechnik GMBH, Wohlerstrabe, Mainz, Germany) with an injection volume of 50 µL. The mobile carrier consists of methanol/acetonitrile (30:70, *v*/*v*) and 10 mM phosphate buffer solution, pH 8. The mobile phase was pumped at a flow rate 1.0 mL/min, and harmine was detected at *λ_max_* = 330 nm”.

### 3.2. Chemicals and Reagents

“All reagents were prepared using de-ionized water (specific resistance = 18.2 MΏ cm) obtained with a Pall-Cascada laboratory water system. Harmine hydrochloride (purity > 98%), high molecular weight PVC, tetradodecylammonium tetrakis (4-chlorophenyl) borate (ETH500), poly (3,4 ethylenedioxythiophene)/poly-(styrenesulfonate) (PEDOT/PSS), and o-nitrophenyl octyl ether (o-NPOE) were obtained from Sigma-Aldrich (St. Lois, MO, USA). Tetrahydrofuran (THF), dioctylphthalate (DOP), tributylphosphate (TBP), acrylamide (AA), ethylene glycol dimethacrylate (EGDMA) and benzoylperoxide (BPO) were purchased from Fluka Chemika-Biochemika (Ronkonkoma, NY, USA). All other chemicals were of analytical grade and used as received without any prior treatment. All solutions were prepared using 40 mM Britton–Robinson (BR) buffer solution. The pH of the buffer solution was adjusted to pH 6 using 0.2 M NaOH. Harmine standard solution (10 mM) was prepared in de-ionized water and stored at 4–6 °C in the refrigerator. All harmine working solutions were freshly prepared from the stock solution daily by appropriate dilution with BP buffer at pH 6. The liquid junction potentials and the activity coefficients were corrected according to the Henderson and Debye–Huckel equations, respectively”.

### 3.3. Biomimics Synthesis

“The main-tailored beads were prepared via a thermal polymerization process. A 1.0 mmol of HME (templated molecule) and 3.0 mmol of AA as a functional monomer were pre-complexed together for 30 min and then, they were mixed together with 3.0 mmol of EGDMA as a cross-linking reagent and 80 mg of BPO as an initiator. The mixture was placed in a capped-glass bottle and 25-mL acetonitrile as a porgenic solvent was added. All dissolved oxygen was removed by passing a flow stream of N_2_ gas for 15 min. Sonication of the solution for 15 min is enough for complete solution homogenization. The polymer was obtained after heating the reaction mixture for 18 h at 80 °C in an oil. Using Soxhlet extraction, harmine molecules were completely removed using a solution mixture of CH_3_COOH/CH_3_OH (1:9, *v*/*v*). The obtained nano-beads were washed several times with CH_3_OH and then, they were left to dry. Non-imprinted polymers (NIPs) were also prepared via the same steps but in absence of an HME molecule and under the same conditions”.

### 3.4. Sensors’ Fabrication

“Glassy carbon (GC) disk electrodes (GC, 4-mm I.D.) were pre-treated and polished by 0.3-µm Al_2_O_3_ to obtain a mirror-like surface. The discs were rinsed with de-ionized water, sonicated with ethanol and de-ionized water alternatively and then dried under N_2_ stream. A piece of Polyvinyl chloride (PVC) tube (1 cm length, 5 mm I.D. and 8 mm O.D.) was inserted at the distal end of the GC electrodes. A 20 μL of PEDOT/PSS dispersant solutions was drop-casted above the GC disk. After the solvent evaporation, PEDOT/PSS coated solid-contact ion-selective electrodes (SC-ISEs) were washed with de-ionized water and then dried under a stream of N_2_ gas. A 100 µL volume of the membrane cocktail was drop-casted on the PEDOT/PSS covered the electrodes, and the solvent was left to be evaporated overnight. The cocktail of the ion-sensing membrane (ISM) (total mass of 110 mg) was prepared by dissolving (10.0 mg) of either MIPs or NIPs beads, (1 mg) ETH 500, (2 mg) potassium tetrakis (4-chlorophenyl) borate, (63.0 mg) o-NPOE, and (34.0 mg) PVC; all in 2-mL THF. Afterward, the membrane was left to dry until a uniform shape was obtained with good adhesion to the GC substrate. All coated-wire electrodes (CWEs) were also fabricated according the above-mentioned steps but without insertion of the PEDOT/PSS transducing-layer. The prepared SC-ISEs were conditioned in 1 mM harmine solution for 4 hrs and then conditioned in 10^−8^ M harmine for 24 h. When not in use, the sensors were kept in the same solution”.

### 3.5. Electrochemical Measurements

“All the pre-conditioned sensors were calibrated by spiking 1.0 mL of 10^−1^–10^−5^ M HME solution in 9-mL BR buffer. The sensors in conjunction with an Ag/AgCl double junction reference electrode were inserted in the test solution and the potential arisen from each harmine concentration was measured. The potential readings were recorded after stabilization to ±0.2 mV and plotted versus log (harmine) concentration. The constructed calibration plots were used for all subsequent measurements”.

Potentiometric selectivity coefficients (*K^Pot^_harmine,B_*) were evaluated using the modified separate solution method (MSSM) [45] and calculated from the equation:

−Log *K^Pot^_harmine,B_* = (*E^o^*_1_ − *E^o^*_2_)/S (1)

where *E^o^*_1_ and *E^o^*_2_ represent the potential readings measured by harmonium ion and the interfering ion at the extrapolated calibration curve (a = 1 M), respectively, and *S* is the slope observed for the primary cation.

Time recovery was evaluated after continuous reading of the potential during the concentration range from 10^−7^ to 10^−2^ M harmine hydrochloride solutions. Effect of pH on the potentiometric response was also investigated after measuring the potential of the proposed sensor in 10^−3^ M harmine solution and changing the pH of this solution from 2 to 10.

Impedance measurements were performed in 10 mM harmine hydrochloride solution within the frequency range 0.01–10^5^ Hz using 0.01 V amplitude at open circuit potential 0.2 V. The working electrodes were the studied electrodes, the reference electrode was Ag/AgCl (saturated KCl), and Pt wire was used as the auxiliary electrode. Chronopotentiomtric measurements were also performed through the three-electrode cell and a reversed current of the value ±1 nA was applied to the working electrode for 60 s as previously described by Bobacka’ s protocol [46].

### 3.6. Assessment of Harmine in Urinary Specimens

Different urine samples were collected from two different volunteers. After collection, samples were stored untreated at −20 °C until further analysis. The samples were spiked with stock solution of harmine. Each 0.1 mL of fresh urine sample was taken and diluted to 10-mL of Britton–Robinson buffer at pH 6 and then directly analyzed.

## 4. Conclusions

In this work, we designed reliable, robust, cost-effective solid-contact potentiometric electrodes for harmine detection. The trace-level assessment of harmine was achieved by integrating a man-tailored artificial receptor for harmine as an ionophores and PEDOT/PSS as a lipophilic solid-contact material. The observed recommended features of PEDOT/PSS as an ion-to electron transducer were reinforced by these studies for designing solid-contact ISEs. The electrodes exhibited a fast-response towards harmine with a Nernstian slope of 59.2 ± 0.8 mV/decade (*n* = 5, *r*^2^ = 0.9996) over the linear range of 1.0 × 10^−7^–1.0 × 10^−2^ M and a detection limit of 0.02 µg/mL. Reasonable selectivity over different common organic and inorganic ions, good accuracy, and possible interfacing with automated systems were presented. These results demonstrated that PEDOT was a promising solid-contact ion-to-electron transducer material in the development of harmine-ISE. The electrodes manifested enhanced stability and low cost that provides a wide number of potential applications for pharmaceutical and forensic analysis. The sensor was successfully applied in monitoring harmine in different urine specimens. The analytical results were compared and agree fairly well with those obtained by gas–liquid chromatography.

## Figures and Tables

**Table 1:** Performance potentiometric characteristics of harmine ISEs.

Parameter	C/Harmine-ISE			C/PEDOT:PSS/Harmine-ISE (o-NPOE)
	o-NPOE	DOP	TBP	
Slope (mV/decade)	58.0 ± 0.7	50.1 ± 0.3	45.2 ± 0.6	59.2 ± 0.8
Correlation coefficient (*r*^2^)	0.9997	0.9994	0.9995	0.9996
Detection limit (µg/mL)	0.03	0.23	0.68	0.02
Linear range (M)	6.0 × 10^−7^–1.0 × 10^−2^	4.0 × 10^−6^–1.0 × 10^−2^	7.0 × 10^−6^–1.0 × 10^−2^	2.0 × 10^−7^–1.0 × 10^−2^
Working pH range (pH)	3–7.5	3–7.5	3–7.5	3–7.5
Response time (s)	<10	<10	<10	<10
Repeatability (% mV)	0.9	1.2	1.1	0.6
Reproducibility (% mV)	1.3	1.1	0.9	0.8
Accuracy (%)	99.1	98.8	98.4	99.6

**Table 2:** Effect of solvent polarity on the selectivity behavior of harmine based sensor.

Interfering Ion	Log K^pot^_Harmine,J_ + SD *		
	o-NPOE	DOP	TBP
K^+^	−5.4 ± 0.2	−5.4 ± 0.3	−5.4 ± 0.1
Na^+^	−5.7 ± 0.3	−5.7 ± 0.2	−5.7 ± 0.1
Harmaline	−1.3 ± 0.2	−1.1 ± 0.1	−0.9 ± 0.2
Strychnine	−3.3 ± 0.1	−3.2 ± 0.1	−3.0 ± 0.2
Caffeine	−3.7 ± 0.2	−3.5 ± 0.3	−3.2 ± 0.3
Atropine	−3.4 ± 0.4	−3.1 ± 0.4	−2.9 ± 0.6
Quinine	−3.8 ± 0.3	−3.7 ± 0.2	−3.6 ± 0.1
Ephedrine	−3.6 ± 0.2	−3.5 ± 0.1	−3.5 ± 0.2
Adrenaline	−3.2 ± 0.3	−3.1 ± 0.4	−3.0 ± 0.3
Glycine	−3.9 ± 0.2	−3.8 ± 0.3	−3.7 ± 0.6

* ±Standard deviation is calculated from three measurements.

**Table 3:** The selectivity coefficients (Log *K^Pot^_Harmine,J_*) of C/PEDOT:PSS/harmine-ISE.

Interfering Ion	Log *K^Pot^_Harmine,J_* ± SD *		
	^a^ C/PEDOT:PSS/harmine-ISE (o-NPOE Plasticizer)	^b^ Harmine/Tetratphenyl Borate-ISE (TBP Plasticizer) [30]	^b^ Harmine/Reineckate-ISE (DOP Plasticizer) [30]
K^+^	−5.5 ± 0.1	−3.6	−3.1
Na^+^	−5.6 ± 0.2	−2.8	−3.2
Harmaline	−1.4 ± 0.1	−0.1	−0.1
Strychnine	−3.4 ± 0.2	−2.0	−1.2
Caffeine	−3.8 ± 0.1	−1.8	−2.0
Atropine	−3.6 ± 0.3	−1.6	−1.8
Quinine	−3.9 ± 0.2	−1.3	−1.1
Ephedrine	−3.5 ± 0.3	−1.9	−1.9
Adrenaline	−3.1 ± 0.5	−1.6	−1.3
Glycine	−4.0 ± 0.1	−3.5	−2.9

^a^ MSSM: Modified separate solution method. ^b^ SSM: Separate solution method.* ±Standard deviation of three measurements.

**Table 4:** Harmine assessment in spiked urine specimens using C/PEDOT:PSS/harmine-ISE.

Sample No.	Spiked Amount, µg/mL	Found, µg/mL				*t*-Student Test	*F*-Test
		Potentiometry	Recovery ^a^ (%) ± SD	HPLC	Recovery ^b^ (%) ± SD		
1	2	1.9 ± 0.3	95 ± 0.5	2.1 ± 0.2	105 ± 1.1	2.7	6.1
2	5	5.1 ± 0.2	102 ± 0.4	4.9 ± 0.3	98 ± 0.3	2.1	4.6
3	10	9.4 ± 0.3	94 ± 0.3	10.1 ± 0.2	101 ± 1.2	3.4	4.5
4	20	20.3 ± 0.2	101.5 ± 0.2	19.6 ± 0.3	98 ± 0.4	1.2	3.2

^a^ Mean of three replicate measurements ± standard deviation (SD). ^b^ *t*-Student and *F*-test at 95% confidence level values are 4.30, 19.00 respectively.

## Data Availability

Data are available according to MDPI Research Data Policies.

## Sample Availability

Samples are available from the authors.
